# The Institutional Worker

**Published:** 1913-01-18

**Authors:** 


THE
### INSTITUTIONAL WORKER
Being a Special Supplement to "The Hospital"

### BUREAU OF INFORMATION.

### Rules for Correspondents.

1. Every letter, on whatever subject, must be accompanied by the coupon to be cut from the back cover (inside page) of The Hospital, current issue, and must contain the name and address of the correspondent with pseudonym for publication if desired.

2. Replies by post cannot be given, save under exceptional circumstances at the Editor's discretion.

3. Letters from Approved Homes in reply to special needs published in the Bureau should state terms and full particulars, and be sent prepaid under cover to the Editor of the Bureau with name written across coupon for identification.

4. Proprietors of Homes which have not yet been entered on the List of Approved Homes, but have spare accommodation likely to suit special needs, are invited to write for an application form for registration. The fee for registration, which includes two announcements of the Home in the Bureau, is 5s.

5. All communications to be addressed to the Editor of The Hospital, 28 Southampton Street, Strand, London, W.C., and marked "Bureau of Information."

**INVITATION TO READERS.—The Editor is prepared to forward to applicants, without fee on either side, offers of accommodation from Homes on the Approved List suitable for medical and surgical cases, for the aged, for chronic or mental patients, for electrical treatment and massage, for convalescents and for children, at varying terms, in any required locality. Reduced terms can often be arranged. The special needs of any who may be sick or in difficulties are also made known in these columns free of charge.**

### Approved Homes Recently Added to List.

Note.—*No Home is added to the List without medical and other testimony to its good management.*

**Bexhill-on-Sea.**—16 Parkhurst Road. Principal: Mrs. W. Hayman. Mental, epileptic, and chronic cases. Husband mental nurse and masseur. Home faces south, with sea view. Near station.

**North Wales.**—Berwynfa, Llangollen. Principal: Miss Edward. Maternity cases, chronic and convalescent. Kindest care and home comforts. Sunny situation and fine air.

One of the principals on the Approved List is obliged to give up her small Home, and would like to correspond with one in the West or South of England, where she could be received in return for services, introducing one chronic patient at moderate terms. Roman Catholic. Can any of our readers assist? Prepaid letters to Charity.

### Miscellaneous.

**Aid for Consumptives.**—Can any of our readers supply the name of the fund which sends consumptives out to St. Moritz and keeps them there at low fees? Also the name and address of the secretary in England, and any other particulars.

**Best Kind of Fire Escape.**—We understand that your hospital has three storeys, that the patients occupy the ground floor and the second, while nurses and maids are on the third floor. We most strongly advise against the proposal you mention to construct a lift from the first to the second floor, with an iron ladder from the second to third floor through a trap door. Such an arrangement might easily prove a death-trap. Nothing is really safe but an outside stairway, which must be separate from the building and able to stand alone. There is a form with a ladder which lets itself down automatically on opening a gate by breaking a glass bar. Stairways at both ends of the building would be the ideal. Apply to the St. Pancras Ironworks, London, N.W., or to Merryweathers, 63 Long Acre, London, W.C., either of which firms would send a representative with the latest information. It will be cheaper as well as more satisfactory to have good advice from an expert, who must, of course, see the place to be of any real help.—Miss E. I.

**Spare Copies of "The Hospital."**—We are pleased to let it be known that you have unbound copies of The Hospital from October 1911 to December 1912 which you are willing to give away. Prepaid replies forwarded to E. D.

### Training and Employment.

**How to Become a School Inspector.**—As you are a trained nurse, we think the post you want must be that of school nurse under the Educational Authorities. These posts are often advertised in our own columns, in other medical papers, and in the *London County Council Gazette*. Training in a children's hospital is an essential qualification, and some knowledge of district work and social conditions is helpful in obtaining posts. Have copies of your testimonials typed, wait for vacancies, and apply as they occur.—Nurse S., Sidmouth.

**Dispensing.**—Unless you are prepared to take a thorough course of training you had better leave pharmacy alone. After three years' training, either with a chemist or a good dispensary, the Minor Examination of the Pharmaceutical Society can be taken. The course, including fees for examination, costs about £100. Salaries vary from £40 to £120 a year for fully qualified pharmaceutical chemists. Write to Miss Brooks, 8 Hunter Street, London, W.C.—M. A. W.

**Massage in Male Nursing.**—Unquestionably as a male nurse you would find a massage certificate well worth the expense and time expended in obtaining it. There is a general preference for masseurs among male patients requiring this treatment, but it takes some time to work up a profitable connection.—Male Nurse.

**Training for Male Probationers.**—Unfortunately there are no civil hospitals except the one you mention where a regular course of training can be procured. But the best training of all is given in the Army Hospitals, and can be obtained by enlisting in the Royal Army Medical Corps. Candidates must be between eighteen and twenty-five years of age. The course is full of varied interest.—S. M., Colchester.

### Nursing in the Empire and Abroad.

**Nursing in Suez.**—We fear that as you have only a midwifery certificate and no general training you would not be suitable for work in this place. The cases likely to be encountered are fevers, together with general medical and surgical work. The resident European population is not large enough to support a maternity nurse alone.—Kitty W.

### Letter-Box.

**Communications have been received and attended to from:**— Nurse R. M., Bray; Miss L. A. F., Boscombe; Miss M. M., Putney.

**The Editor begs to acknowledge the receipt of communications, with enclosures, from:**— Mrs.

W., Broadstairs; Mrs. A., Catford; Miss L. A. F., Boscombe.

**The Editor is much indebted for testimonials received in response to inquiries from:** — Miss A. Steleman; Mrs. Bass; Miss Furrell; Miss Garrett; W. Silverwood Richardson, F.R.C.S.

**Letters have been forwarded from:**—Miss A. B.; Mr. M. R.; Mrs. D.

We are greatly obliged to you for your kind letter offering help. We have sent it on and asked Edwards to communicate with you direct.—SPES.

It is impossible to forward letter, as rules were not complied with.—MRS. GOUGH.

We are making full inquiries, and will write more fully next week.—THE REV. F. M. L.

The Editor is unable to introduce patients to any but the Homes on the Approved List.—F. REED.

### Institution Facts and Figures.

### Selected Answers to December Questions.

THE MOST POPULAR SUPPER DISH.

WE find that the supper dish most appreciated by our staff is *Savoury Stew*, made as follows:—8 lb. of mutton or beef pieces, 14 lb. of potatoes, 6 lb. of onions, three bunches of carrots, ½ lb. of dripping, twopennyworth of turnips or parsnips, seasoning, cold water. Layers of meat and vegetables are put into large cookers on the range, with seasoning and small pieces of dripping among the layers. The latter is not used if the meat is fat, but our pieces are usually the opposite. The cookers are filled half-full with cold water and closely covered. The cooking required is from four to seven hours, according to the size of the cooker. It must be slow or the meat will be tough. This is one of our winter supper dishes, and we reckon that our cold nights and strenuous work renders a good hot supper very essential. Cost is usually 4s. 4d. for fifty persons, about a fraction over 1d. per head. If the contract price for carrots and turnips is high for any reason, they are left out and more onions or celery used.—MATRON, North Country.

We never supply meat for supper in this institute and find no one ever grumbles on this point. Perhaps *Macaroni Cheese* is the prime favourite, no one ever seems to tire of it. The quantities are: 7 lb. of straight macaroni, 3 lb. of cheese (stale bits usually), 6 quarts of skimmed milk, 1 lb. of dripping, 1 lb. of flour, made mustard, salt, pepper. Break up the macaroni, throw it into fast boiling salted water, and boil it for about 45 minutes, or till soft. Then drain it well. Melt the dripping in a pan—we generally use two pans in order to boil the sauce more speedily—mix in the flour smoothly, and pour in the milk. Stir now and then until the sauce boils. Grate or grind the cheese, add three-quarters of it to the sauce, and season it with salt, mustard, and pepper after adding the macaroni. Turn the mixture into greased deep tins and bake in the oven until well browned. Cost, 4s. 6d. for fifty persons; per head, about 1d.—SISTER D.

MILK SUPPLY.

(*a*) Quantity used in women's surgical ward, 15 gallons; (*b*) in women's medical ward, 19 gallons; average number daily in each case, 13. Diets are calculated according to dietary scale by ward sister and given to matron or her assistant with notes of extras as—*e.g.*, tuberculous patient on so many pints over and above that carried by diet. Milk measured (under supervision) by dairyman directly into ward cans, which are sent at once to wards. When patient has been ordered more milk than he can take sister is expected to report this and order accordingly. Average cost in surgical ward per head, 9d.; in medical ward per head, 11½d.—"JOHN."

A LARGE PROVINCIAL HOSPITAL.

*Consumption of milk in medical and surgical wards during a given week of December* 1912:—

| Ward | Consumption for Week | Average Daily Number of Patients | Average Quantity per Patient per Week | Contract Price per Gallon | Cost of Patient per Week |
|---|---|---|---|---|---|
| | gall. pt. | | gall. pt. | d. | s. d. |
| Male Surgical | 26 7 | 26 | 1 0¼ | 9½ | 0 9·8 |
| Female ditto | 26 2 | 28 | 0 7½ | 9½ | 0 8·9 |
| Male Medical | 36 6 | 23 | 1 4¾ | 9½ | 1 3·1 |
| Female ditto | 38 0 | 25 | 1 4⅙ | 9½ | 1 2·4 |

The ordering of milk by the ward sister is on the following basis:—Half-pint of milk per day for each patient on ordinary diet; one pint of milk per day for each patient on milk diet; two pints of extra milk for each milk diet patient on extra milk, making three pints per day for such patient. The above figures show very clearly that more milk is used in the medical than in the surgical wards. This is, of course, due to the fact that there are fewer patients on ordinary diet in the medical wards and more on extra milk.—"INSTITUTIONAL."

QUESTIONS FOR JANUARY.

1. State the method in use in your institution for making either beef-tea or broth, or both. How much does the liquor work out at per pint respectively? State the price paid for the meat, and what saving, if any, is effected by the use of a stock-pot.

2. What difference could you make in the bill of fare for the staff, irrespective of their numbers, if you were allowed an extra shilling per head per week for their board? The original allowance may be estimated at six, seven, or eight shillings per head. It is desired to demonstrate how much could be obtained in luxuries for the extra shilling.

The figures must be the result of actual observation and computation, not estimates. Either or both of the questions may be answered by each contributor. The best answers will be published, and for each contribution considered by the Editor to be of sufficient interest for publication payment of FIVE SHILLINGS will be made.

The following rules must be observed by contributors to the FACTS AND FIGURES Column:—

1. Answers must be written on one side of the paper. They must bear the name and address of the sender and be accompanied by coupon to be cut from the back cover (inside page) of the current issue of THE HOSPITAL. A pseudonym must be chosen if the name is not to be published.

2. Answers must be addressed to the Editor of THE HOSPITAL, 28-29 Southampton Street, Strand, London, W.C.; they must be marked in the left-hand corner "Facts and Figures," and must be received before the end of the month.

### Editor's Notices.

**Contributions:**

Contributions should be written, or preferably typed, on one side of the paper only, and all articles sent in are accepted upon the distinct understanding that they are forwarded to The Hospital only.

The Editor cannot undertake to return MSS. not used, but when a stamped directed envelope is enclosed the MSS. may be returned if a special request is made.

Accepted articles and paragraphs of news will be paid for after publication at the scale rate.

**Address:**

To prevent delay all contributions and letters on editorial business must be addressed exclusively to the Editor, "The Hospital," 29 Southampton Street, Strand, London, W.C. It is important that this regulation shall be strictly observed.

**Correspondence:**

Correspondence on all subjects is invited. The name and address of correspondents must be given as a guarantee of good faith, but not necessarily for publication.

**Special Articles:**

Special articles are invited, and questions, inquiries, and paragraphs upon all matters relating to the work, administration, and management of general, special, mental, fever, cottage and convalescent hospitals, sanatoria, homes, institutions, societies and organisations for the treatment or care of the sick, injured and dependants of all classes. Every matter affecting the interests and welfare of the staff of all grades working in these institutions will receive special consideration.

**Administrative Medicine, Research and Items of News:**

Special terms will be paid for approved articles on subjects in this wide field by experts who have made some section of it their special study and interest. We wish to give prominence to every movement tending to advance accuracy of diagnosis, efficiency in treatment, and the development of modern methods to eradicate disease and promote the welfare of the suffering.

**A Bureau of Information:**

We invite inquiries and applications for information and help in providing for that numerous class of sufferers who require special care or house-room which they cannot obtain in their own homes or secure for themselves. **We cannot, however, prescribe, or recommend practitioners.**

**Books for Review:**

Publishers are particularly requested to send advance proofs of any new books of importance whenever possible, as the Editor has made arrangements to publish immediate reviews on a new plan.

**Photographs, Plans, Blocks, and Illustrations:**

It is requested that wherever possible MSS. may be accompanied by illustrations in any of the above forms. The name of the sender and of the article to which it belongs should be written on the back of each photograph, drawing, or block for purposes of identification.

**Local Papers:**

Newspapers containing reports or news paragraphs should be marked and addressed to the Sub-Editor.

**The Coupon System:**

A coupon will be found at the bottom of the third inside page of the cover each week. This coupon must be attached to every question or inquiry to which an answer is desired in The Hospital.

### Sale and Exchange.

Doubtless you possess articles that are no longer in use but still in a serviceable condition. Why not turn these into money by disposing of them through our Private Sale and Exchange Columns? This section is reserved exclusively for bargains between our Readers, and privacy in all transactions is assured by the employment of box numbers, *all replies being forwarded through* The Hospital *Offices*.

Moreover, by utilising our "Deposit System" Readers can with perfect safety forward goods on approval to any part of the country.

Announcements in this section cost 1s. for 14 words and 6d. for 7 words or part of 7 words beyond.

If you have something to sell—some article which is not in use—do not let it rust or rot, but give some other Reader an opportunity of securing it through the medium of The Hospital Private Sale and Exchange Columns.

Sale and Exchange Announcements, 14 words ... ... 1s.

| | | |
|---|---|---|
| | | |
| | | |
| | | |
| | | |
| | | |
| | | |
| | | |

### Manager's Notices.

**Letters relating to the publication, sale, and advertising departments of "The Hospital" must be addressed to The Manager, "The Hospital," The Hospital Building, 28 and 29 Southampton Street, London, W.C.**

**Subscriptions** may begin at any time, and are payable in advance. Cheques and Post-Office Orders should be crossed London County and Westminster Bank, Covent Garden Branch, and made payable to the Manager of The Hospital.

Rates of Subscription (including Postage).

| *United Kingdom.* | | *Foreign and Colonial.* | |
|---|---|---|---|
| Three Months ... ... | 2s. 0d. | Three Months ... ... | 2s. 6d. |
| Six Months ... ... | 4s. 0d | Six Months ... ... | 5s. 0d. |
| Twelve Months ... ... | 6s. 6d | Twelve Months ... ... | 8s. 8d. |

**Advertisements:**

To ensure insertion, all advertisements for The Hospital must reach the Manager not later than Wednesday morning in each week. For scale of charges see pages v and 4.

**Remittances:**

**It is especially requested that remittances be made by Cheque or Postal Order, and in halfpenny stamps only when the amount is under sixpence.**

